# SCALES FOR MEASURING AGEISM AS EXPERIENCED BY OLDER ADULTS: LITERATURE REVIEW AND METHODOLOGICAL CRITIQUE

**DOI:** 10.1093/geroni/igz038.921

**Published:** 2019-11-08

**Authors:** Hyun Kang

**Affiliations:** University of Kansas, Lawrence, Kansas, United States

## Abstract

A growing body of research shows that ageism negatively affects older adults’ psychological well-being and even physical functioning. However, the tools to measure ageism as experienced by older adults are not well developed. This study reviewed the literature on ageism scale with an emphasis on the methodological issues. Most standardized ageism scales have focused on younger people’s attitudes and beliefs toward older adults. We found only one standardized scale that examined how older adults felt and thought about their experiences being treated as a stereotype. However, the scale is incomplete because it does not fully measure ageism and it has received far less rigorous analysis. Many studies have adopted and revised ageism scales that were developed specifically to measure younger people’s attitudes toward older adults, meaning that the scales’ validity has been problematic when administered to older adults. Furthermore, many studies that discussed older adults’ experience of ageism used uni-dimensional or simple measures. Although significant efforts have been made to outline ageism’s various dimensions and constructs, these efforts have not led to a common consensus on ageism and its characteristics. Lack of consensus, in turn, makes it harder to develop a standardized scale. Finally, existing scales are more suitable for Western societies. Socio-cultural uniqueness has not been considered when developing scales, nor has the scales’ cross-cultural reliability and validity been tested. Our findings suggest that a new scale that applies only to measuring ageism as perceived by older adults and corresponds to the significant dimensions of ageism must be developed.

